# The Clinical Conundrum of Vasoplegia With Mechanical Circulatory Support Devices∗

**DOI:** 10.1016/j.jacadv.2024.100914

**Published:** 2024-03-23

**Authors:** Mandeep R. Mehra, Francesco Castagna

**Affiliations:** Center for Advanced Heart Disease, Brigham and Women’s Hospital and Harvard Medical School, Boston, Massachusetts, USA

**Keywords:** cardiac surgery, LVAD, vasoplegia, vasopressors

**Figure:**
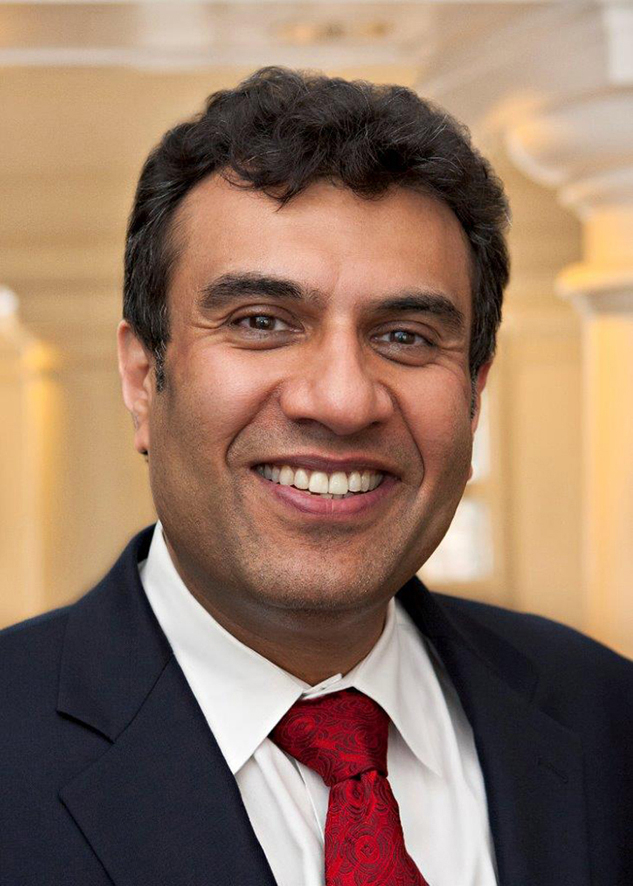

The recognition of damaging effects of cardiopulmonary bypass, known as “postperfusion syndrome,” marked a significant early milestone in cardiac surgery. Evidence surfaced, suggesting systemic inflammation, coagulopathy, hemolysis, multisystem organ failure, and vasoconstriction as notable consequences, each leading to deleterious outcomes. However, a distinct syndrome began to emerge in the early 1990s, characterized by preserved cardiac output, low to normal filling pressures, and systemic hypotension unresponsive to fluid resuscitation.^1^ This syndrome, different from postperfusion syndrome, manifested as intense vasodilation, tissue hypoperfusion, and cellular dysfunction. Vasoplegia, as it came to be known, demanded high doses of catecholamines for correcting the low systemic vascular resistance and was often accompanied by oliguria, metabolic acidosis, and a high mortality rate. Progress in understanding the pathophysiology of this entity came through elucidating vasomotor tone determinants, endothelial dysfunction, and neurohormonal regulation in vascular homeostasis. Experimental models, such as endotoxin-induced shock in animals, shed light on inflammatory pathways and cytokine cascades implicated in vasoplegia.^2^ Its multifactorial underpinnings included oxidative stress-related endothelial dysfunction, dysregulation in renin-angiotensin-aldosterone system and endothelin, upregulation of adenosine, nitric oxide (NO), cyclic guanosine 3′,5′-cyclic monophosphate, increases in prostacyclin, and vasopressin deficiency.^3^

In this issue of *JACC: Advances*, Lamba et al^4^ discuss their single-center experience with vasoplegia during durable continuous flow left ventricular assist system implantation. While their evaluation primarily concerns legacy devices, the findings likely hold for contemporary systems. Device-specific flow characteristics or surgical techniques for novel devices may not necessarily contribute to postoperative vasoplegia. They identify a high incidence of vasoplegia in nearly a third of patients, primarily through a liberal definition encompassing both short (lasting <12 hours) and prolonged vasoplegia (lasting >12 hours), with the former being predominant. Nested analyses reveal that short-duration vasoplegia carries lower morbidity and outcomes like those encountered in patients without vasoplegia. Prolonged vasoplegia, lasting beyond 12 hours, bears a malignant prognosis, associated with a greater likelihood of renal failure and death, albeit occurring in only 13% of cases. Risk factors for prolonged vasoplegia include increased body mass index and preoperative renal failure, with the prolonged duration of cardiopulmonary bypass (CPB) and worse liver dysfunction as adjudicated by an elevated Model for End-Stage Liver Disease scores nearing significance thresholds (with lower boundary 95% CIs of 0.99). These argue against our ability to significantly modify this risk preoperatively suggesting that our concentration on therapeutic strategies that may serve to mitigate and treat development of vasoplegia in the intraoperative and perioperative period are more worthy of clinical consideration.

Despite the lack of confirmatory evidence, clinicians employ several practices to limit the incidence of post operative vasoplegia. These tactics include withholding beta-blockers (inconsistent association) and renin-angiotensin-aldosterone system inhibitors (through their bradykinin enhancing effect), avoiding use of perioperative inotropic-vasodilator drugs such as the phosphodiesterase-3 inhibitor milrinone (through smooth muscle relaxation), and reducing the use of drugs such as amiodarone (which may mediate their negative effects through thyroidal dysregulation) are considered as candidate risk mediators for vasoplegia.^5^ None of these practices have been conclusively established and generally are directed towards clinician comfort. One area of increasing activity relates to transition from temporary to durable mechanical circulatory support. In such cases, we believe that the risk for vasoplegia may be heightened due to ongoing hemolysis and oxidative stress with a consequent systemic inflammatory response and endothelial dysfunction with devices such as trans-aortic micro axial flow pumps or extracorporeal membrane oxygenation systems often used in support of circulations as a prelude to definitive surgical therapy.^6^ In such cases, the intraoperative use of CPB should be limited as far as possible and early targeted treatment of vasodilatory shock may be useful. In some circumstances, the use of systemic hemoadsorption devices designed to remove circulating cytokines has been suggested as a useful tactic, but this too remains largely anecdotal. This device, akin to dialysis systems can be added to the CPB circuit and includes a cartridge containing biocompatible copolymer beads capable of removing molecules of medium molecular weight by using a combination of hydrophobic or ionic interactions as well as hydrogen bonding. Studies, mostly in sepsis syndromes, have been of low yield although this technology was utilized in dealing with the hyperinflammation syndrome observed during the COVID-19 pandemic; however, most studies have not shown a benefit to support widespread use.^7^

Once vasoplegia becomes manifest, and after filling pressures have been adequately optimized, use of catecholamine and non–catecholamine-based drugs are necessary, often in high doses. However, it is not known nor well studied what sequence or combination of such drugs ought to be employed for successful outcomes. While this represents an area for scientific inquiry, until such evidence can be forthcoming, clinicians have employed distinctive tactics, but their effects on outcomes are uncertain. The primary mode of treatment uses catecholamines, preferably norepinephrine, which has been shown to be associated with the least hemodynamic and physiological stress in this clinical scenario. As an example, in comparative effectiveness studies, norepinephrine has fewer toxic effects compared to dopamine, and in other shock states epinephrine is associated with greater expression of lactic acidosis than norepinephrine.^8^^,^^9^ A useful strategy to limit high doses of catecholamines posits use of concomitant vasopressin, an agent that exploits a known deficiency of this hormone in this situation and one that in combination with catecholamines may avoid the deleterious tissue effects of high doses of a single agent.^10^^,^^11^ Vasopressin (or its analogs) predominantly acts by binding to the arginine vasopressin receptor A1 (with other effects on oxytocin) which in turn suppresses cyclic guanosine 3′,5′-cyclic monophosphate activation due to NO.^10^ Other noncatecholamine drugs include angiotensin II (which acts via vasoconstrictive effects of the angiotensin type 1 receptor on smooth muscles, increases in sympathetic drive and also influences vasopressin release from the posterior pituitary), methylene blue (which suppresses NO and cytokine cascade effects), hydroxycobalamine (which acts as a NO scavenger) and the “HAT” combination which includes hydrocortisone, ascorbic acid, and thiamine.^12^ The hypothesis of “HAT” use is based on the demonstration of marked vitamin C deficiency due to CPB as well as in the potential restorative function of this combined supplementation on endothelial disruption. However, most studies in vasodilatory shock (mostly due to sepsis) have not shown benefit and such use remains experimental or anecdotal.^13^^,^^14^

The study of vasoplegia warrants more thoughtful integration of prevention and treatment strategies to mitigate its negative pathophysiological consequences. Standardized nomenclature and identification of precise pathophysiological targets are crucial for progress. As Confucius said, *“There is never a case when the root is in disorder and yet the branches are in order.”*

## Funding support and author disclosures

Dr Mehra has received consulting fees derived from Abbott (paid to his institution), Natera, Paragonix, Moderna, NupulseCV, Fineheart, Transmedics, Leviticus, Cadrenal, and Second Heart Assist. Dr Castagna has reported that he has no relationships relevant to the contents of this paper to disclose.
